# Supplementary figures and images for: Accuracy and repeatability of the Microsoft Azure Kinect for clinical measurement of motor function
Source: PLoS One. 2023 Jan 26;18(1):e0279697. doi: 10.1371/journal.pone.0279697 (PMC9879399; doi:10.1371/journal.pone.0279697)

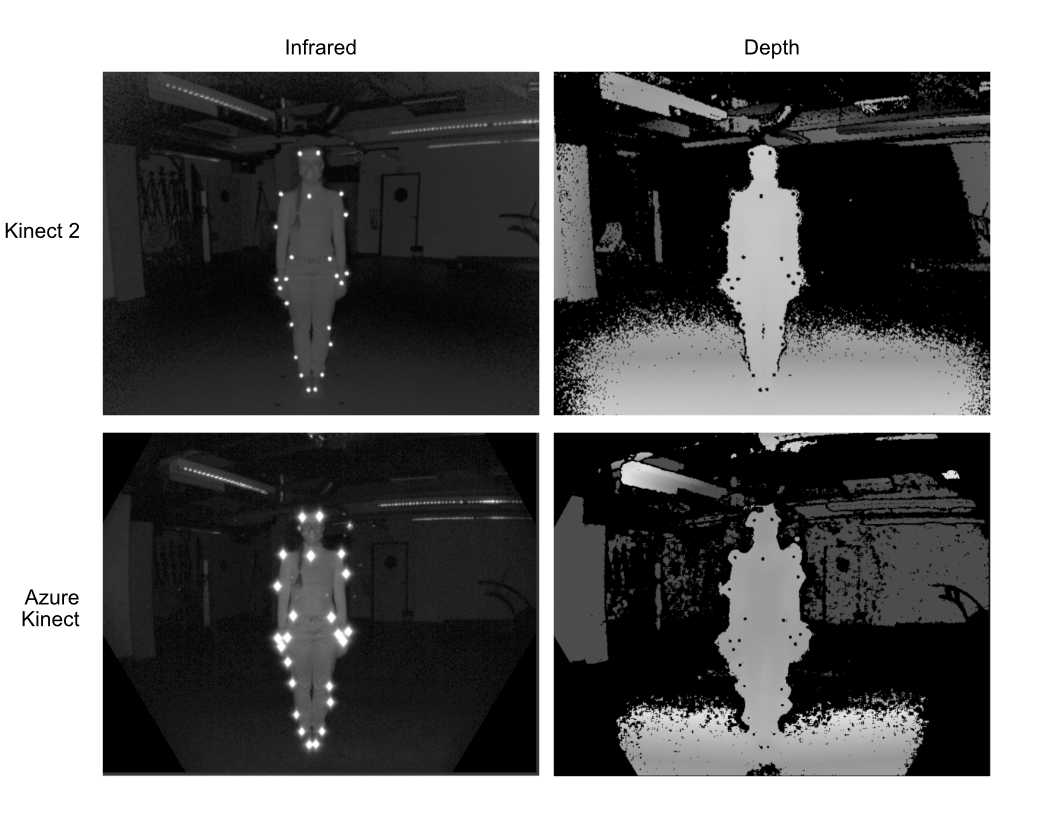

Supplement: S1 Fig — The recording angle slightly differs between both sensors, resulting in more pronounced distortions of the walls in Azure Kinect images. (TIF) [file pone.0279697.s003.tif]

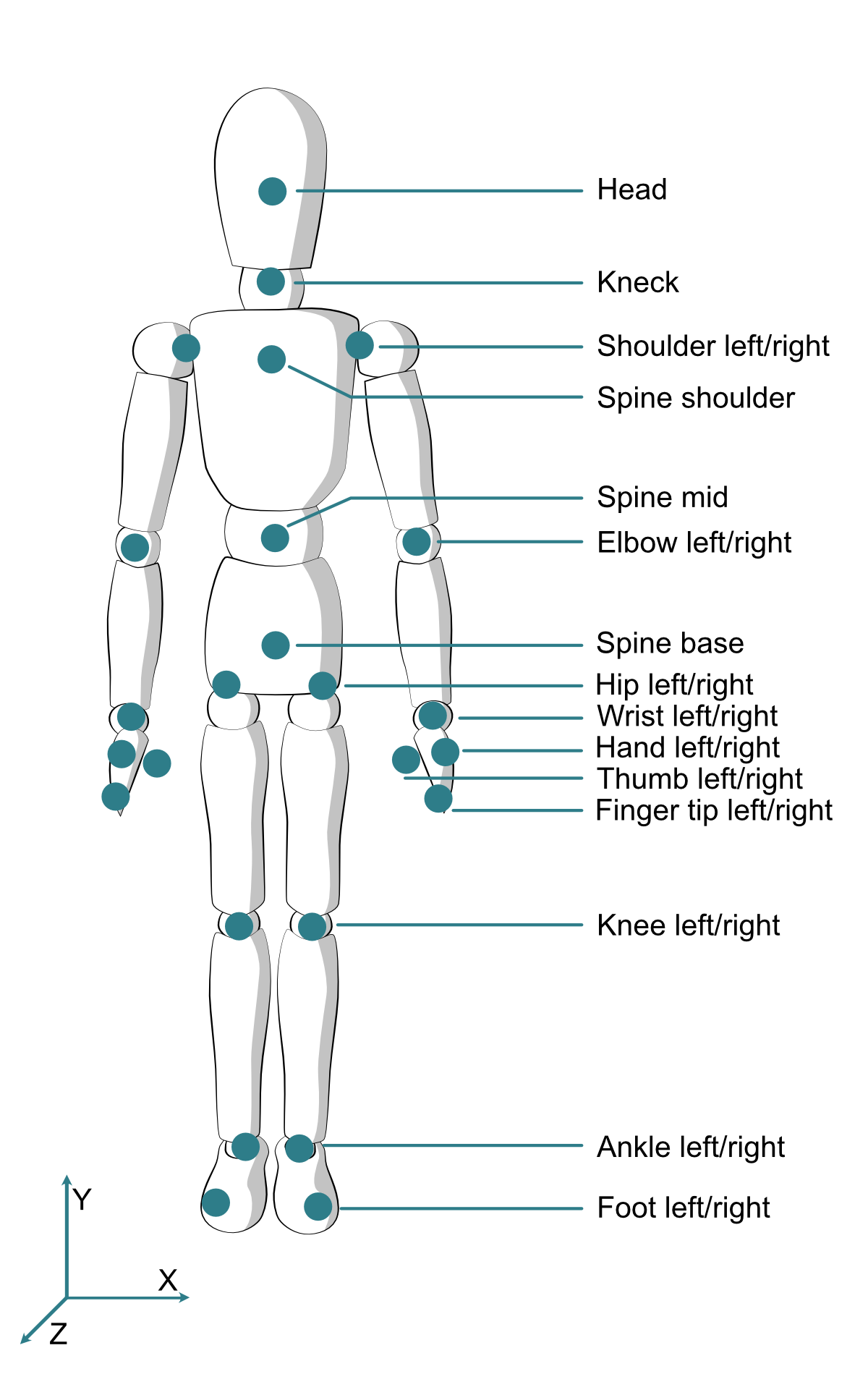

Supplement: S2 Fig — (TIF) [file pone.0279697.s004.tif]

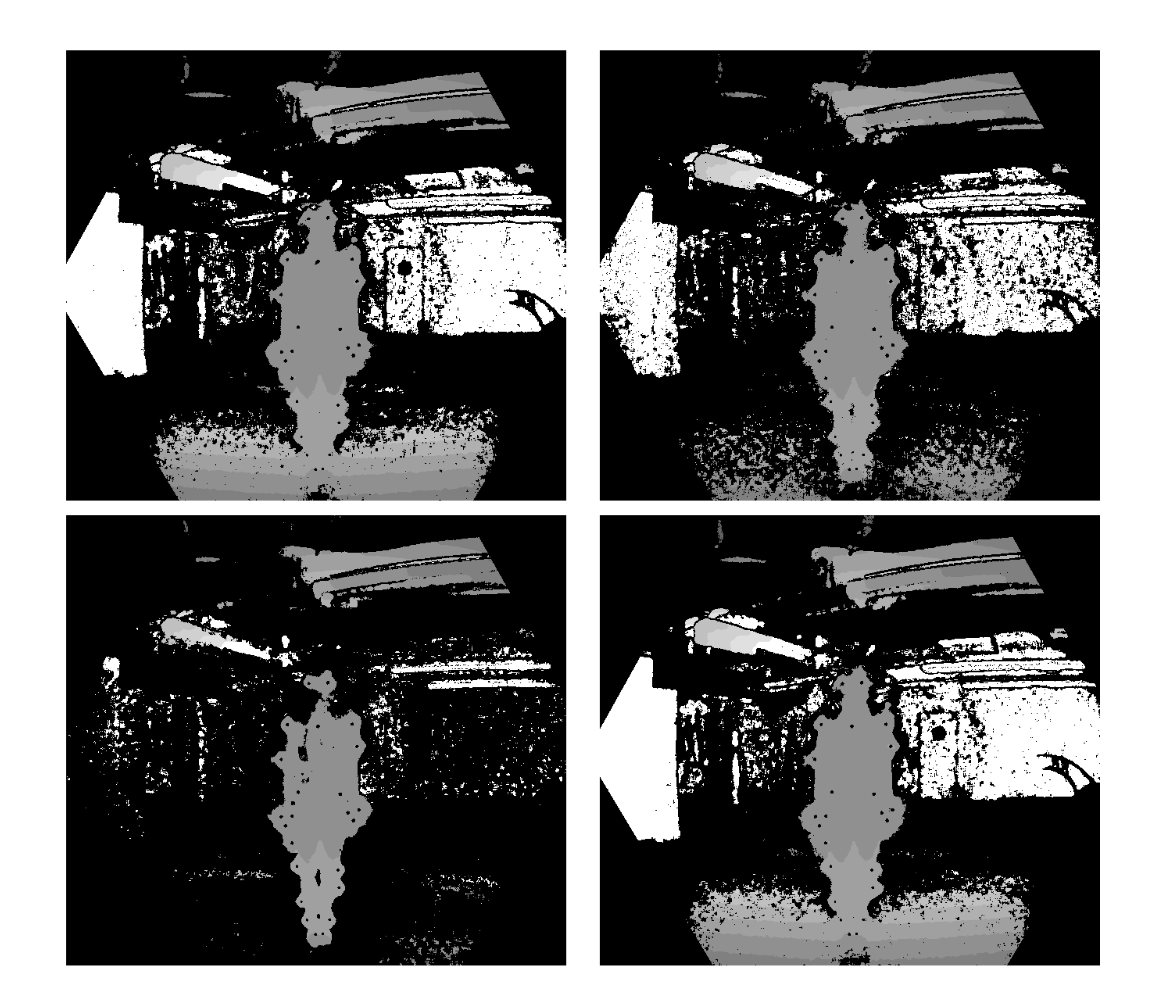

Supplement: S3 Fig — (TIF) [file pone.0279697.s005.tif]

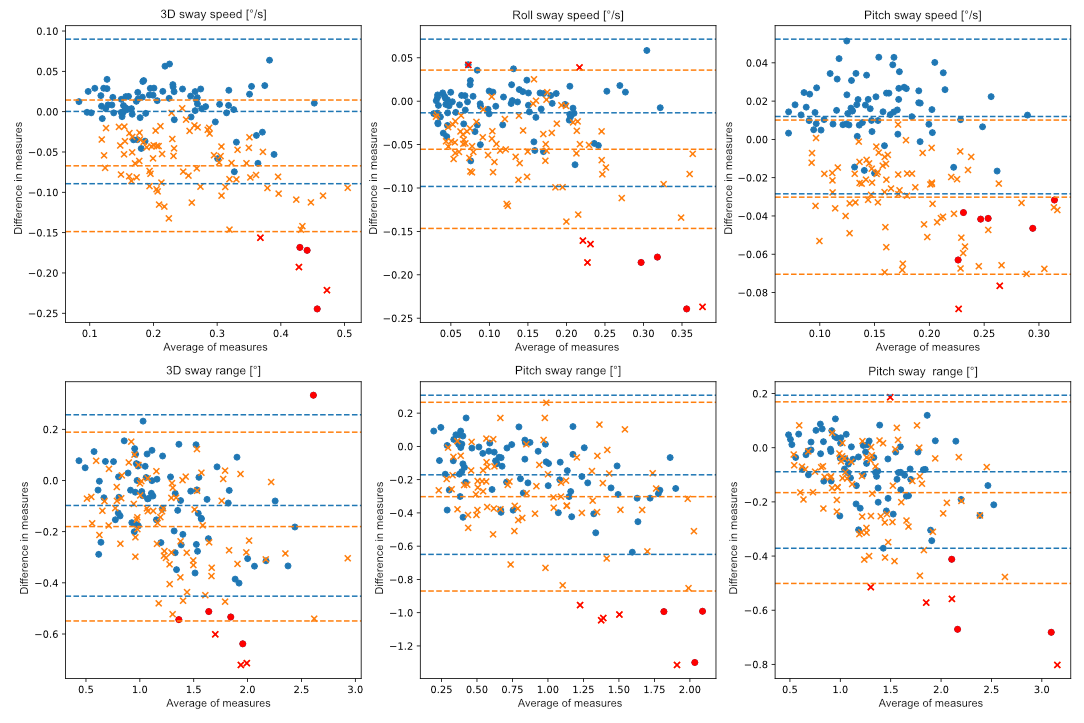

Supplement: S4 Fig — Outliers above and below the Limit of Agreement (LOA) are marked red. (TIF) [file pone.0279697.s006.tif]

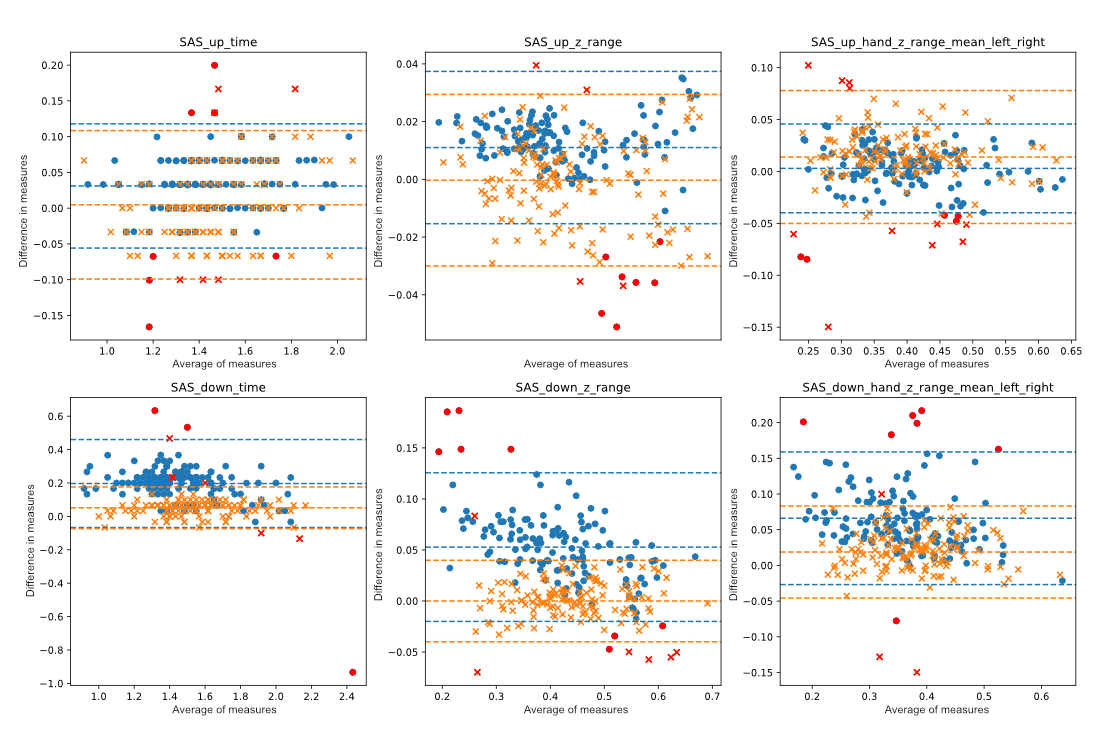

Supplement: S5 Fig — Outliers above and below the Limit of Agreement (LOA) are marked red. (TIF) [file pone.0279697.s007.tif]

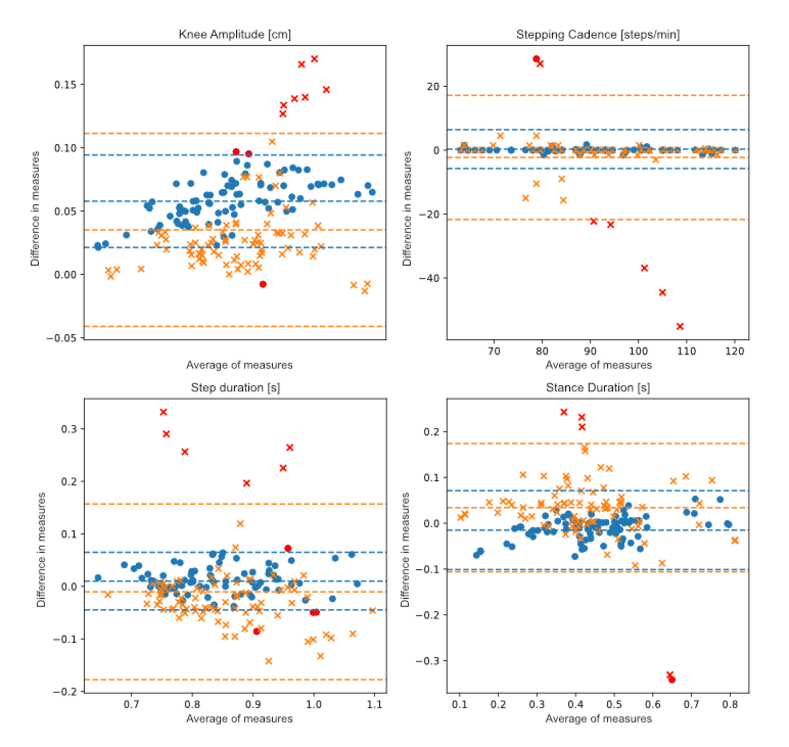

Supplement: S6 Fig — Outliers above and below the Limit of Agreement (LOA) are marked red. (TIF) [file pone.0279697.s008.tif]

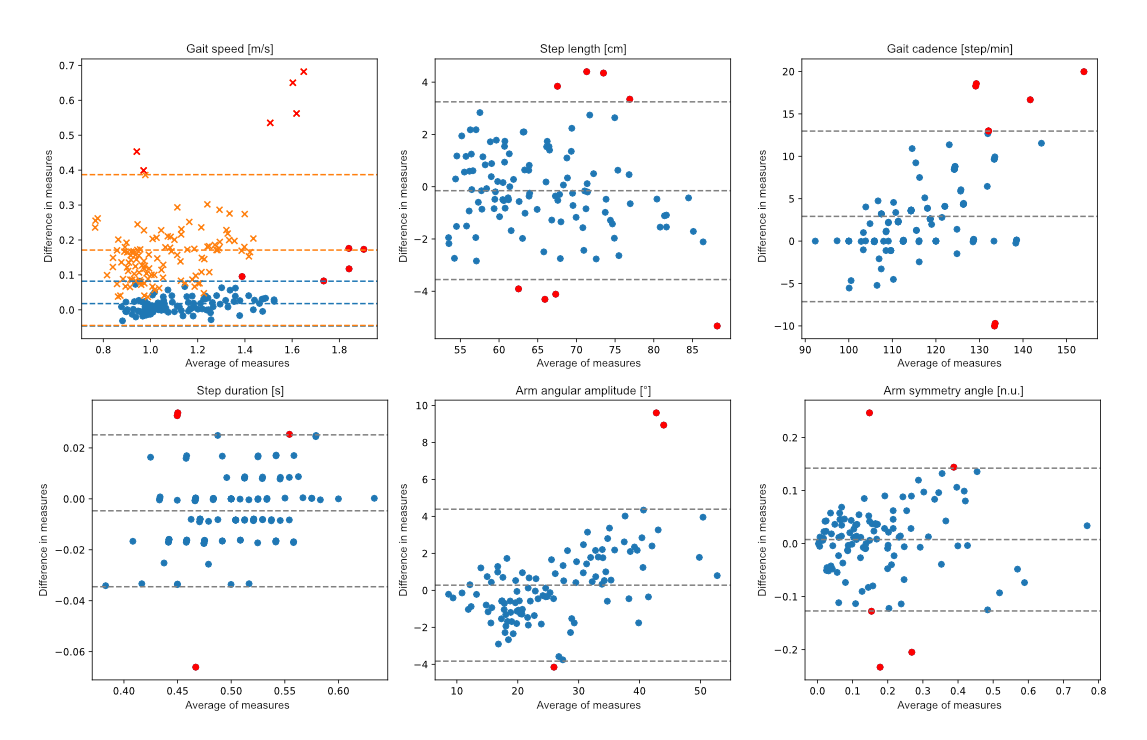

Supplement: S7 Fig — Outliers above and below the Limit of Agreement (LOA) are marked red. (TIF) [file pone.0279697.s009.tif]
