# Supplementary material for: Accuracy and repeatability of the Microsoft Azure Kinect for clinical measurement of motor function
Source: PLoS One. 2023 Jan 26;18(1):e0279697. doi: 10.1371/journal.pone.0279697 (PMC9879399; doi:10.1371/journal.pone.0279697)
Supplement: Attachment — Submitted filename: 2022-11-03 Feedback from Editor and Reviewers clean.docx [file pone.0279697.s014.docx]

We would like to thank both reviewers for their time and valuable feedback. Please find attached our detailed responses to you feedback below.

**Feedback from Reviewer #1:** Dear Authors.

The work is very interesting and supports the results of others previously published with the Kinect One camera that this system is an easy solution for the evaluation of movement. The only problem is in relation to the sample size and the non-evaluation by age range.

The authors should explain the limitations of the sample and the age of the participants that prevent them from demonstrating the strength of the results.

Answer to the feedback from the authors:

Throughout the manuscript, we strengthened the fact that the cohort consists of young healthy participants and advanced the statement regarding the study limitations. (see line 298ff).

**Feedback from Reviewer #2:** This manuscript is well done and achieves its aim to assess the agreement between a marker system (the ‘gold’ standard Qualysis) against a newer marker-less system, and their respective reliability.

I have several observations which should be strongly considered before publication.

1. I am uncertain of the role of the K2 in this study. There is no convincing argument for including this device and comparing it to the others, especially when (as the authors state in at least 2 sentences) the K2 is no longer manufactured. If the K2 has never been compared to a 'gold standard' (which is implied on line 61,) it's a bit late!. Having these data also complicates the agreement and reliability analysis.

Answer to the feedback from the authors:

**Thank you for your feedback in this regard. Our wording in line 61 seems to have been unclear, so we have rewritten the sentence. The Kinect version 2 (K2) has been used for a variety of clinical trials in recent years and, despite being discontinued, is still very much in use for research purposes as recent publications show (eg, Ma et al. Kinect V2-Based Gait Analysis for Children with Cerebral Palsy: Validity and Reliability of Spatial Margin of Stability and Spatiotemporal Variables. Sensors (Basel). 2021 Mar 17;21(6):2104). The camera and its pose estimation software was extensively investigated regarding their technical and clinical feasibility previously. And in some clinical studies, the K2 was even used as the only measurement device for motor function, without any additional clinical-reference system. The new generation of sensors has just entered this testing phase regarding its usability, which is why we think it is of importance and an actual strength of our manuscript to not only provide marker-based reference measures but also results from the K2. In this way, readers and researchers can compare their existing study results based on the K2 and can estimate potential differences to the new sensor system. This is in our opinion one of the important information required for switching to the K4.**

2. The statistical analysis is well done and comprehensive. However, when assessing the level of agreement between the systems (and hence the newer system’s (i.e. AK) validity against the ‘gold’ standard) the authors have opted for ICC (A,1). I strongly expect most readers will be also expecting to see the more familiar **Bland and Altman plots** to assess levels of agreement. Even though the ICC (A,1) does this, the visual effect of the Bland and Altman plots are important.

Answer to the feedback from the authors:

**We created the Bland-Altman plots and added them as supplementary figures 4 to 7.**

3. The second piece of advice surrounds the phrase ‘gold standard’. A more appropriate term, in this study using these systems, would be ‘**clinical reference standard’**. This means that it is the best available method, rather than the perfect method which the phrase ‘gold standard’ often implies.

Answer to the feedback from the authors:

**We changed the phrasing throughout the whole manuscript.**

4. Technical points. Figures 2 & 4 were unhelpful. Not sure what readers are supposed to see in this. This was even after I downloaded the tiff files.

Answer to the feedback from the authors:

**We moved these figures to the supplementary material. Our reasoning against a deletion of these figures is, that for researchers familiar with these systems the information is quite valuable. Either to compare the image resolution as well as to get an idea how these inferences would look like in the recorded depth data.**

**Typographical errors**

Line 57 spelling ‘these’

Line 57-62: it makes more sense to have the verbs in the past tense (i.e. extended, determined)

Line 68-69: This makes more sense to say that these were “exclusion criteria”

Line 76: “day- and artificial light“ ; delete the hypen

Line 114: insert ‘were terminated’

Line 303 “subtle disease-“ delete the hyphen

Line 307: it’s better to write more simply: “which is no longer manufactured”; I would repeat this version in the conclusion on line 328.

Line 325: The word ‘current’ here is actually a little confusing as at first glance it seems it should be ‘concurrent validation experiments’ (which I presume it is not). It would be better to write: “ In conclusion, the present validation experiments….”

Answer to the feedback from the authors:

**We fixed all typographical errors provided. Thank you again very much for your support and valuable feedback to our manuscript.**
